# Exploring the Role of Th10 Cells and IL-10 in Systemic Lupus Erythematosus

**DOI:** 10.7759/cureus.63875

**Published:** 2024-07-05

**Authors:** Shradha Verma, Seema Shah, Rachita Nanda, Jhasaketan Meher, Vinay Rathore, Suprava Patel, Eli Mohapatra

**Affiliations:** 1 Biochemistry, All India Institute of Medical Sciences, Raipur, Raipur, IND; 2 General Medicine, All India Institute of Medical Sciences, Raipur, Raipur, IND; 3 Nephrology, All India Institute of Medical Sciences, Raipur, Raipur, IND

**Keywords:** correlation, lupus nephritis, th10 cells, interleukin-10, systemic lupus erythematosus

## Abstract

Introduction: Systemic lupus erythematosus (SLE) is an autoimmune disease characterized by autoantibody production and immune complex deposition in various organs. The pathogenesis of SLE is multifactorial, involving genetic, hormonal, environmental, and immune factors. Interleukin-10 (IL-10) is a pleiotropic cytokine produced by various immune cells and has conflicting roles in inflammation.

Materials and methods: This is a cross-sectional study involving 56 SLE patients and 30 healthy controls.

Results and analysis: We found a significant increase in T helper 10 (Th10) cells and IL-10 levels in SLE patients compared to controls. Disease activity, measured by Systemic Lupus Erythematosus Disease Activity Index (SLEDAI) score, correlated positively with Th10 cells and IL-10 levels. Further analysis categorized patients into active and inactive SLE, showing significant differences in laboratory parameters, including C3, C4, Th10 cells, and IL-10, between the two groups. Notably, Th10 cells and IL-10 exhibited a significant positive correlation with SLEDAI scores. The study also explored SLE patients with and without nephritis, a severe manifestation of the disease. Th10 cell expression was significantly higher in nephritis patients, while IL-10 levels did not differ significantly between the two groups.

Conclusion: In conclusion, this study provides valuable insights into the association between Th10 cells, IL-10, and disease activity in SLE. The findings suggest that Th10 cells and IL-10 could serve as potential biomarkers for disease activity in SLE, offering a basis for further research into therapeutic interventions targeting these factors. These results contribute to our understanding of the complex immunological factors at play in SLE and may pave the way for more targeted and effective treatment approaches.

## Introduction

Systemic lupus erythematosus (SLE) manifests as a derangement of the immune system, characterized by dysregulation of B cell activity, leading to the exuberant production of autoantibodies. These autoantibodies subsequently deposit within various organs as immune complexes, triggering tissue damage and inflammation [1]. SLE is multifactorial in pathogenesis and involves genetic, hormonal, environmental, and immune dysregulation; however, the exact mechanism is not known. It is well known that a variety of immune cell types and signaling molecules, or cytokines, play a critical role in the inflammatory response and tissue damage associated with SLE [2]. Interleukin-10 (IL-10), a cytokine with diverse effects, is produced primarily by macrophages, but it is also produced by various other immune cell types, including Th2 cells, T regulatory cells, B cells, cytotoxic T lymphocytes, natural killer cells, mast cells, and dendritic cells. Additionally, neutrophilic and eosinophilic granulocytes contribute to IL-10 secretion [3]. Several studies have shown conflicting results regarding the inflammatory role of IL-10. A study by Ravirajan et al. [4] reported a pathogenic role of IL-10 in causing glomerular immune deposition in severe combined immune deficient (SCID) mice. Studies by El-Fetouh et al. [5] have shown a positive correlation between elevated levels of interleukin-10 (IL-10) and interleukin-18 (IL-18) and both the Systemic Lupus Erythematosus Disease Activity Index (SLEDAI) score and disease severity in SLE patients. Similarly, a study in Han Chinese patients showed a high IL-10 level, which was correlated with other inflammatory markers and anti-double-stranded DNA (anti-dsDNA) and anti-nucleosome antibodies [6]. However, Ling et al. [7] observed a protective effect of IL-10 against lupus development in a mouse model, where dendritic cells were implicated in disease induction. The contradictory role of IL-10 may be explained on the basis of its dual role. IL-10 stimulates antibody production by stimulating B cells; however, its effect on T cells is inhibitory in nature, so it has a diametrical role in inflammation [1].

IL-10 is primarily produced by Th10 cells. The designation of cells as Th10 cells is applied to the IL-10-producing subset of CD4+ T helper cells, irrespective of the origin of CD4+ T helper cells. A study by Caielli et al. [8] has shown an increased expression of Th10 cells in blood and tubulointerstitial areas in SLE. In our study, we reported the presence of Th10+ cells and an elevated concentration of IL-10 in lupus patients. Although we could not identify the origin of Th10 cells, whether from natural or inducible T regulatory cells, Th2 or CXCR5, CXCR3+ PD-1hi CD4+ T cells, our study showed a correlation between IL-10 level and disease severity. As of today, limited studies deciphering the role of Th10 and its correlation with disease activity have been done.

## Materials and methods

This cross-sectional study was conducted in the Department of Biochemistry in collaboration with the Department of Medicine of All India Institute of Medical Sciences (AIIMS), Raipur, Chhattisgarh, between 2021 and 2022. Fifty-six SLE patients as per Systemic Lupus International Collaborating Clinics (SLICC) and 30 healthy controls were included in the study, after obtaining informed written consent [9]. The study was approved by the Institutional Ethics Committee of AIIMS, Raipur (AIIMSRPR/IEC/2020/515). Patients having viral hepatitis, diabetes mellitus types 1 and 2, thyroid disorders, and other autoimmune diseases or if pregnant were excluded from the study. Disease activity was measured clinically as per the total SLEDAI score.

Samples were collected under aseptic conditions in heparinized vacutainers. Flow cytometric analysis was done on whole blood within 24 hours of blood collection, followed by separation of plasma by centrifugation at 3,000 rpm for 10 minutes. Separated plasma was stored at -80 degrees centigrade until the estimation of IL-10.

Flow cytometric analysis of T helper 10 (Th10) cells was done in a three-laser, 10-color Beckman Coulter Navios flow cytometer with incorporated Navios software version 1.2. The following monoclonal antibodies supplied by Thermo Fisher Scientific were used: anti-CD45 Pacific Orange, anti-CD3 fluorescein isothiocyanate (FITC), anti-CD4 allophycocyanin (APC), anti-CCR-6 (super bright 436), anti-IL-17 phycoerythrin (PE), and anti-IL-10 PE-Cyanine7. Two test tubes were used: one for the test and another for the isotypic controls. Whole blood (100 μL) was added to each tube. For RBC lysis, 2 mL of 1× RBC lysis buffer was added and incubated for 10-12 minutes at room temperature. It was followed by washing two times with staining solution by vortexing and centrifugation until a clear cell pellet was obtained. Freshly prepared Foxp3 Fixation/Permeabilization solution (1 mL) was then used to fix all the cells uniformly by vortexing. It was then centrifuged, and the supernatant was discarded. This was followed by staining of cells with the cocktail of antibodies (5 μL each): CD45, CD3, CD4, and IL-10 in the test tube. In the control tube, no antibodies were added. Incubation in the dark for 20 minutes was then done at room temperature, followed by washing with staining solution. Lastly, the pellet was resuspended in 500 μL of staining solution, and flow cytometric analysis was done.

Based on forward and side scatter properties, a singlet population was separated. Lymphocytes were gaited with the help of CD45 and side scatter. T helper cells were identified as double positive for CD3 and CD4. A subset of T helper cells was found to be IL-10-positive and labeled as Th10 cells. Th10 was identified by double positive status for surface antibodies CD3, CD4, and IL-10 intracellularly. To rule out the nonspecific binding of monoclonal antibodies and the effect of autofluorescence, isotype-matched controls were used.

Plasma IL-10 was measured by sandwich ELISA (human IL-10 by Bioassay Technology Laboratory, Zhejiang, China, with linearity of 5-1,500 pg/mL and sensitivity of 2.59 pg/mL).

Statistical analysis

The initial data capture was performed in a spreadsheet (Microsoft Excel version 2013; Microsoft Corp., Redmond, WA). Subsequently, all statistical analyses were conducted utilizing the SPSS software package version 20 (IBM SPSS Statistics, Armonk, NY). The distribution of data was studied using the Shapiro-Wilk test. Parametric data were presented as mean ± standard deviation (SD) and compared using the independent t-test, whereas non-parametric data were presented as median and interquartile range and compared using the Mann-Whitney U test. A p-value of less than 0.05 was employed as a criterion for statistically significant results.

## Results

Demographic data

The study included 56 cases of SLE and 30 healthy controls. The median age of controls and cases was 27.5 (5) and 27 (9) years, respectively, with no significant difference (p = 0.95). The majority (94.6%) of the cases were females (n = 53) with 5.4% (n = 3) males, with a male/female ratio of 1:9. The median duration of the disease in SLE cases was 3 (2.25) years, ranging from 1-9 years. Disease activity was based on the SLEDAI score, and the median was 4.50 (8).

Routine and immunological parameters

The mean hemoglobin was low in cases (9.36 ± 2.2) as compared to controls (12.6 ± 0.9), and the difference was statistically significant (p < 0.001). No statistically significant difference in total leukocyte count (TLC) values (p = 0.63) was found; however, a significant difference was observed in platelet count (p < 0.001) between controls and cases.

ESR and CRP levels were significantly higher in cases than in controls (p < 0.001 and p = 0.007, respectively). The increase in serum C3 and C4 levels was significant (p < 0.001). ANA was positive in all patients (100%), while anti-double-stranded DNA (anti-dsDNA) was positive in 39.28% of cases of SLE.

**Figure 1:**
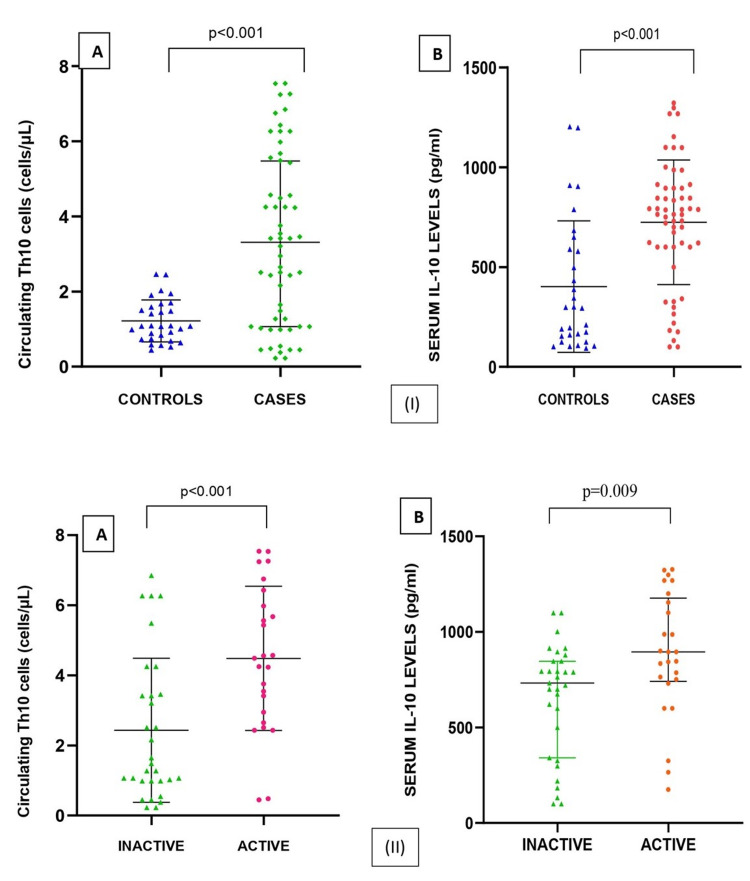
Comparison of circulating Th10 cells and serum IL-10 among (I) controls and cases and (II) inactive and active SLE Th10: T helper 10, IL-10: interleukin-10, SLE: systemic lupus erythematosus

A statistically significant (p < 0.001) increase in the expression of Th10 cells was found among cases (3.43 (4.00)) when compared to controls (1.07 (0.87)). IL-10 level was higher in cases (791 (313)) as compared to controls (296 (457.4)) and was found to be significant (p < 0.001) (Figure 1).

Patients were categorized as active (SLEDAI: ≥6) and inactive (SLEDAI: 0-5), with 25 cases as active and 31 as inactive SLE. The laboratory parameters ESR, CRP, SLEDAI score, C3, C4, Th10, and IL-10 were found to be significantly different in active SLE as compared to inactive SLE, whereas ANA was not (Table 1 and Table 2).

**Table 1:** Demographic characteristics and laboratory parameters for inactive and active SLE patients Data are presented as mean ± SD or median (interquartile range). SLE: systemic lupus erythematosus, Hb: hemoglobin, TLC: total leukocyte count, ESR: erythrocyte sedimentation rate, CRP: C-reactive protein, SD: standard deviation

Parameters	Inactive (n = 31)	Active (n = 25)	p-value
Age (years)	28 (8)	27 (12)	0.76
Females/males (number (%))	30 (96.77)/1 (3.22)	23 (92)/2 (8)	0.43
Duration of the disease (years)	3 (2.50)	2 (2)	0.02
Hb (g/dL)	9.78 ± 2.10	8.83 ± 2.31	0.11
TLC (10^3^/mm^3^)	6.99 (3.13)	5.32 (4.10)	0.13
Platelet (10^3^/μL)	257 ± 72.2	228 ± 113	0.23
ESR (mm/1^st^ hour)	66 ± 41.5	74.8 ± 37.9	0.41
CRP (mg/dL)	13.1 (18.9)	15.3 (17.7)	0.16
Proteinuria (number (%))	8 (25.8)	6 (24)	0.88

**Table 2:** Immunological investigation for inactive and active SLE patients Data are presented as mean ± SD or median (interquartile range). SLE: systemic lupus erythematosus, SLEDAI: Systemic Lupus Erythematosus Disease Activity Index, C3: complement 3, C4: complement 4, anti-dsDNA: anti-double-stranded DNA, Th10 cells: T helper 10 cells, IL-10: interleukin 10

Parameters	Inactive (n = 31)	Active (n = 25)	p-value
SLEDAI score (number (%))	4 (3)	14 (12)	<0.001
C3 (mg/dL)	87.5 ± 12.7	46.4 ± 26.2	<0.001
C4 (mg/dL)	14.8 ± 5.49	11 ± 7.46	0.03
Anti-dsDNA positivity (number (%))	11 (35.4)	11 (44)	0.52
Th10 (cells/μL)	2.16 (3.19)	4.56 (2.96)	<0.001
Serum IL-10 (pg/mL)	732 (504.9)	895 (436)	0.009

**Figure 2:**
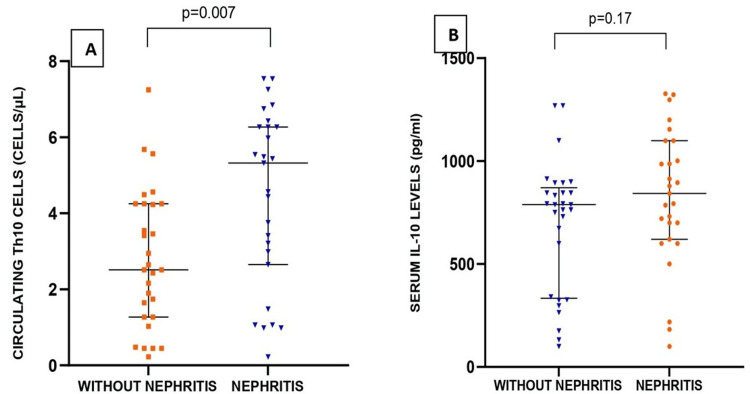
Comparison of circulating Th10 cells and serum IL-10 among SLE patients with and without nephritis Th10: T helper 10, IL-10: interleukin-10, SLE: systemic lupus erythematosus

SLE can affect various organs, and kidney involvement, known as lupus nephritis, is a severe manifestation. Among the recruited SLE patients, 27 cases had SLE nephritis. The median age of patients without nephritis and with nephritis were 29 (9) and 25 (8) years, respectively, with no significant difference (p = 0.21). Similarly, there was no significant difference in the duration of disease (p = 0.06) and SLEDAI scores (p = 0.66) among the two groups. No statistical difference in the levels of hemoglobin (p = 0.22), TLC (p = 0.36), and platelets (p = 0.22) was found between the two groups. However, a statistically significant difference for serum C3 (p < 0.001) and C4 (p = 0.006) was observed between the two groups. Serum ESR (p = 0.59) and CRP (p = 0.41) revealed no statistically significant difference. Similarly, seropositivity for anti-dsDNA (p = 0.07) and proteinuria (p = 0.95) were also not statistically significant. The expression of Th10 cells in patients with nephritis (5.3 (3.61)) was significantly higher than in patients without nephritis (2.51 (2.98)) (p = 0.007). Elevated levels of IL-10 were observed in patients with nephritis (843 (478)) compared to patients without nephritis (789 (536)) and were not significant (p = 0.17) (Figure 2).

A significant positive correlation between both Th10 (r = 0.434, p = 0.001) and IL-10 (r = 0.352, p = 0.008) and SLEDAI was seen.

**Figure 3:**
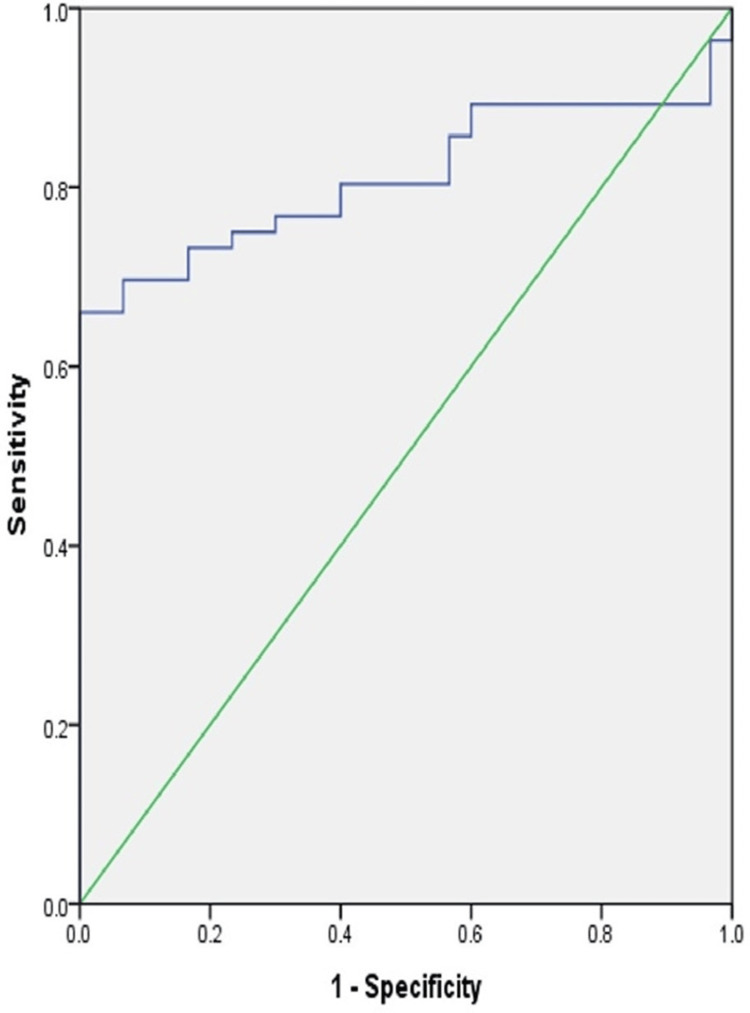
Receiver operating characteristic curve of Th10 in predicting the severity of SLE Th10: T helper 10, SLE: systemic lupus erythematosus

The area under the receiver operating characteristic (ROC) curve for Th10 was measured at 0.811. The cutoff at a value of 1.48 cells/μL showed sensitivity at 76.8% and specificity at 70% with p < 0.001 (Figure 3).

## Discussion

Systemic lupus erythematosus (SLE) serves as a model autoimmune disease characterized by the over-activation of B cells from various lineages, leading to the production of antibodies that target the body's own tissues. These autoantibodies form complexes that deposit in multiple organs, ultimately causing damage [2]. Although the exact pathogenesis remains to be elucidated, several cells and cytokines are considered crucial for pathogenesis, including IL-10. IL-10, a pleiotropic cytokine, is produced by almost all the leukocytes. The influence of interleukin-10 (IL-10) on the development of SLE remains unclear, with studies reporting contradictory findings. IL-10 exhibits seemingly contradictory properties. While it acts as a potent inhibitor of T lymphocytes, suggesting anti-inflammatory effects, it can also promote B cell survival, proliferation, differentiation, and autoantibody production, potentially contributing to inflammation [9,10].

Expression of T helper cells and its subtypes Th2, Th17, and T follicular helper cells is increased in all SLE cases and is accompanied by an increase in serum cytokines such as interleukin-6 (IL-6), interleukin-10 (IL-10), interleukin-17 (IL-17), and interleukin-21 (IL-2). Studies have revealed that T-reg cells maintain self-tolerance in the body by inhibiting autoreactive lymphocytes. This suggests that dysfunction in T-reg cells may be a factor in the pathogenesis of SLE [11].

A new subpopulation of T helper cells expressing CD45+, CD4+, CD3+, and IL-10+ was found in our study, which was designated as Th10 cells. The expression level of Th10 cells was shown to be statistically increased in SLE cases when compared to healthy control with p < 0.001. Similarly, in active SLE cases, the rise in Th10 expression was found to be statistically significant at p < 0.001, signifying an inflammatory action of Th10 cells. The pro-inflammatory role of Th10 was further advocated by a positive correlation coefficient of 0.434 between SLEDAI and Th10, which was found to be statistically significant at p = 0.001.

The expression of circulating Th10 cells was also significantly higher in patients with lupus nephritis as compared to patients without lupus nephritis and was found to be statistically significant at p = 0.007. A higher expression of Th10 in the tubulointerstitial area was also reported by Caielli et al. [8] in lupus nephritis patients. The increased level of circulating Th10 cells in lupus nephritis patients may have been contributed by both circulating Th10 cells and tubulointerstitial tissue.

The area under the ROC curve for Th10 was measured at 0.811. The cutoff at a value of 1.48 cells/μL showed sensitivity at 76.8% and specificity at 70% with p < 0.001. So, at a cutoff of 1.48 cells/μL, the expression of Th10 cells can thus be utilized as an evidence-based immunological biomarker of disease activity in SLE patients, particularly for remote monitoring in secondary or tertiary healthcare facilities, where skilled immunologists may not be available physically.

These Th10 cells produce IL-10, which is markedly increased in SLE patients. The overproduction of IL-10 can be attributed to the immune complex-mediated activation of peripheral blood mononuclear cells (PBMCs) via Fc-γ receptor II, followed by further increased production of IL-10 by Th10 cells [12]. IL-10 promotes B cell survival and differentiation by decreasing its apoptosis through the inhibition of Bcl-2 expression [13]. IL-10, along with succinate, causes the generation of mitochondrial reactive oxygen species (ROS) by reversing the electron transport chain. This synergistic action results in the transformation of naive B cells into antibody-producing plasma cells, thus resulting in B cell survival, proliferation, differentiation, and autoantibody production [8]. The contribution of B cells to autoimmunity goes beyond solely generating plasma cells and autoantibodies; rather, it also acts as an antigen-presenting cell (APC) and produces pro-inflammatory cytokines, as its contribution toward developing autoimmunity.

In our study, the median level of IL-10 was found to be significantly elevated in SLE as compared to healthy control. Further, active SLE patients were shown to have a significant elevation of IL-10 when compared to inactive SLE patients, which was statistically significant at p = 0.009. Similar findings were also reported by various researchers such as Abd Elazeem et al. [12], Godsell et al. [14], Yang et al. [15], Bassiouny et al. [16], and McCarthy et al. [17]. However, Chen et al. [18] and Cigni et al. [19] reported no significant difference. This contradictory finding can be attributed to the genetic and phenotypic variation of the population studied, their treatment status, and different sample sizes.

Non-significant (p = 0.17) elevation levels of IL-10 were also observed in lupus nephritis patients, which was supported by similar findings as reported by Zeid et al. [20] and Sigdel et al. [21]. Zeid et al. [20] also discussed the role of the IL-10 gene (-592 A/C) polymorphism in causing renal involvement in their study.

In our study, we found a positive correlation between SLEDAI score and plasma IL-10 (r = 0.288, p = 0.008). Since a high SLEDAI score implies a higher disease activity, a positive correlation between SLEDAI and plasma IL-10 proposes the use of plasma IL-10 as a marker of disease activity. Our findings were supported by various researchers such as El-Fetouh et al. (r = 0.551, p < 0.01) [5], Abd Elazeem et al. (r = 0.34, p = 0.01) [12], Bassiouny et al. (r = 0.51, p = 0.01) [16], McCarthy et al. (r = 0.324) [17], and Houssiau et al. (r = 0.368, p < 0.005) [22].

This research was the first of its kind to study the correlation of SLEDAI with circulating Th10 cells and IL-10 levels in SLE. However, our study was limited by the small sample size. Since this study was cross-sectional in nature, the pathogenic role of IL-10 could not be established. Also, the immunomodulatory effect of various drugs on the expression of various immune cells and their production of various cytokines at different therapeutic doses was not taken into account. A prospective study on a larger sample size will help in understanding the role of these biomarkers in SLE pathogenesis and could pave the way for improved diagnosis and targeted therapies.

## Conclusions

In conclusion, the expression of peripheral blood Th10 cells and serum IL-10 were found to be increased in SLE patients as compared to controls, with a significant positive correlation with SLEDAI. Future prospective studies on a larger sample size are required to validate these results and for their translational application. Th10 cells and IL-10 can thus be utilized as an evidence-based immunological biomarker of disease activity in SLE patients, particularly for remote monitoring in secondary or tertiary healthcare facilities, where skilled immunologists may not be available physically.
